# Genome-Wide Association Study Uncover the Genetic Architecture of Salt Tolerance-Related Traits in Common Wheat (*Triticum aestivum* L.)

**DOI:** 10.3389/fgene.2021.663941

**Published:** 2021-05-20

**Authors:** Xiaoyan Quan, Jindong Liu, Ning Zhang, Chunjuan Xie, Hongmei Li, Xianchun Xia, Wenxing He, Yuxiang Qin

**Affiliations:** ^1^Department of Biological Science, School of Biological Science and Technology, University of Jinan, Jinan, China; ^2^Agricultural Genomics Institute at Shenzhen, Chinese Academy of Agricultural Sciences, Shenzhen, China; ^3^Institute of Crop Sciences, National Wheat Improvement Center, Chinese Academy of Agricultural Sciences, Beijing, China

**Keywords:** candidate gene, GWAS, Na/K, QTL, salt tolerance, wheat

## Abstract

Soil salinity is a serious threat to wheat yield affecting sustainable agriculture. Although salt tolerance is important for plant establishment at seedling stage, its genetic architecture remains unclear. In the present study, we have evaluated eight salt tolerance–related traits at seedling stage and identified the loci for salt tolerance by genome-wide association study (GWAS). This GWAS panel comprised 317 accessions and was genotyped with the wheat 90 K single-nucleotide polymorphism (SNP) chip. In total, 37 SNPs located at 16 unique loci were identified, and each explained 6.3 to 18.6% of the phenotypic variations. Among these, six loci were overlapped with previously reported genes or quantitative trait loci, whereas the other 10 were novel. Besides, nine loci were detected for two or more traits, indicating that the salt-tolerance genetic architecture is complex. Furthermore, five candidate genes were identified for salt tolerance–related traits, including kinase family protein, E3 ubiquitin-protein ligase-like protein, and transmembrane protein. SNPs identified in this study and the accessions with more favorable alleles could further enhance salt tolerance in wheat breeding. Our results are useful for uncovering the genetic mechanism of salt tolerance in wheat at seeding stage.

## Introduction

Soil salinity is a serious abiotic stress affecting more than 800 million hectares of total agricultural land worldwide (Song and Wang, 2015; Ding et al., 2018; Li W. H. et al., 2020). Plant growth and development, including seed germination, shoot height (SH), root length (RL), and biomass, are significantly inhibited under salt stress (Hasan et al., 2017; Liu et al., 2018; Füzy et al., 2019). It continues to be a serious threat to agricultural sustainability as greater than 50% of agricultural land will be salinized by 2050 because of unscientific irrigation management, such as flood irrigation (Flowers, 2004; Demiral and Türkan, 2006). Hence, developing salt-tolerant cultivars is one of the most effective and sustainable ways to utilize the saline alkali land.

Salt stress damages plant tissues through osmotic stress and ionic toxicity to cells (Zhu, 2003; Munns and Tester, 2008). Osmotic stress is the first stress to confront when plants are subjected to salt stress, because salt stress first decreases root water-uptake ability by reducing soil water potential (Liang et al., 2018). Subsequently, Na^+^ at high concentrations is absorbed by the roots and transported to the shoots. Excessive cellular Na^+^ has a negative effect on metabolic processes and photosynthetic efficiency (Munns, 2005; Ahmadi et al., 2011), and it also makes it difficult to maintain intracellular ion homeostasis due to impeding K^+^ absorption (Munns and Tester, 2008; Shabala and Cuin, 2008). Both osmotic stress and ion toxicity cause oxidative damage of membrane lipids and proteins and further disturb plant growth rate and development (Mittler, 2002; Imlay, 2003).

Plant salt tolerance is controlled by a series of quantitative trait loci (QTLs) or genes and is a typically genetic and physiological trait (Flowers, 2004; Munns and Tester, 2008). The complexity of mechanisms involved in salt stress tolerance for plants limits the progress toward salt-tolerance breeding. Mapping the QTLs for salt tolerance–related traits and breeding cultivars with high salt tolerance have become an effective way to reduce the losses caused by salt threat (Akram et al., 2020). QTL mapping based on biparental populations is a traditional approach to dissect the genetic mechanisms of complex quantitative inheritance traits (Kumar et al., 2015). However, only two allelic effects in any single locus can be evaluated in a biparental mapping, which limits its power to uncover the nature of genetic variation in wheat (Shi et al., 2017). Thus, the genome-wide association analysis (GWAS) method, which is based on linkage disequilibrium (LD), has become an alternative way to identify markers significantly associated with complex agronomic traits (Mackay and Powell, 2007; Cockram et al., 2010; Wang et al., 2014; Winfield et al., 2015; Liu et al., 2017; Akram et al., 2020), biotic stress (Singh et al., 2020; Dababat et al., 2021), and abiotic stress (Rehman-Arif and Börner, 2020). Compared with traditional biparental mapping, GWAS is more efficient and less expensive with no need to develop biparental population and a more representative gene pool. More recently, GWAS has become an effective tool for dissecting the genetic architecture of salt tolerance–related traits in crops such as wheat (Liu et al., 2017), rice (Kumar et al., 2015), barley (Long et al., 2013), and soybean (Kan et al., 2015).

Land salinization has become a serious threat in China because of the increased irrigation management, climate change, and fertilizer use. Breeding for salt-tolerant cultivars could be greatly improved by the identification and application of molecular markers. Furthermore, plants are the most sensitive to salinity at seeding stage, other than flowering, and the grain filling stage (Gerona et al., 2019). Thus, it is of great significance to identify loci related with salt tolerance at seedling stage of wheat. We investigated a diverse panel of 317 elite wheat cultivars employing GWAS to (1) uncover the genetic mechanism of salt tolerance, (2) detect the markers associated with salt tolerance, and (3) search the candidate genes and accessions with more favorable alleles for salt tolerance.

## Materials and Methods

### Plant Materials and Treatments

To evaluate the salt tolerance of the modern cultivars, 317 various wheat accessions, mainly including modern cultivars and improved accessions, comprising 260 accessions from China, and 57 accessions from other countries (Supplementary Table 1) were used for GWAS. The experiment was carried out in a growth chamber at 20 ± 2°C with a photoperiod of 16-h light/8-h dark and a light intensity of 300 μmol m^–2^ s^–1^. Seeds of all the accessions were germinated and grown to 7-day-old seedlings. The uniform seedlings were transplanted into 20-L containers covered with polystyrol plates with 60 evenly spaced holes. Half-strength modified Hoagland’s solution was used for the cultivation of the seedlings with continuous aeration (Wu et al., 2015; Shan et al., 2018). The cultivation solution was renewed every 5 days. Completely randomized experiment was designed with four replicates, involving 16 plants grown separately. Each experiment comprised eight randomized units allocated to control (0 mM NaCl) and salt (200 mM NaCl) treatment groups. Two-leaf seedlings were treated with 200 mM NaCl; NaCl was gradually added with a 100-mM increment per day.

### Phenotypic Measurement and Data Analysis

The plants were harvested for measuring SH, RL, shoot fresh weight (SFW), and root fresh weight (RFW) after 1 week of salt treatment. Shoots and roots were separated after washing with distilled water and oven-dried at 105°C for 30 min and then dried further at 80°C for 72 h. Record shoot dry weight (SDW) and root dry weight (RDW). And then 100 mg fine powder of dried shoot was incubated with 5 mL extraction buffer in a 90°C water bath for 30 min. The extraction buffer was a mixture of 60% trichloroacetic acid, nitric acid, and sulfuric acid (2:10:1). The supernatant was taken after centrifugation. Na^+^ and K^+^ content was estimated using atomic absorption spectrophotometer (TAS-990).

Analyses of variance (ANOVA) of all the tested traits among genotypes and replicates were determined with SAS v9.3 (SAS, Institute, http://www.sas.com), and the differences at *P* < 0.01 were considered highly significant. The salt-tolerance index was employed the relative changes of SDW, RDW, SFW, RFW, SH, RL, and shoot K content (labeled as RSDW, RRDW, RSFW, RRFW, RSH, RRL, and RK, respectively), as well as the values of Na content and Na/K ratio under salt stress (Munns and James, 2003; Long et al., 2013). The relative change of each trait was calculated by the value of salt stress/control, for example, RSDW = SDW (salt)/SDW (control). Basic statistical analysis of the data of all the phenotypic traits under both treatments and all the salt-tolerance indices was performed using Microsoft Excel 2019. Pearson correlations of the salt-tolerance indices of 317 wheat accessions were conducted in SPSS version 16.0.

### Genotyping and Population Structure Analysis

All the 317 accessions were genotyped with the wheat 90 K single-nucleotide polymorphism (SNP; Illumina, 81,587 SNPs) chip by Capital Bio^1^. SNPs used for subsequent GWAS analysis were obtained after quality control (minor allele frequency > 0.05 and missing data < 20%; Liu and Muse, 2005). The physical positions of SNPs were obtained from the International Wheat Genome Sequencing Consortium website (IWGSC, http://www.wheatgenome.org/; IWGSC v1.1).

Population structure was analyzed using 1,000 filtered SNPs with Admixture 1.3.0 program^2^. ADMIXTURE ran from *K* = 2 to *K* = 12 clusters to identify the optimal *K* value. Principal components analysis (PCA) with a number of five components and phylogenetic trees [neighbor-joining (NJ)] were also used to uncover the population information with the software GAPIT (Lipka et al., 2012) based on R 3.5.3 and Tassel v5.1 (Bradbury et al., 2007), respectively. Besides, the LD was estimated using GAPIT software (Lipka et al., 2012) according to Liu et al., 2017.

### Genome-Wide Association Analysis

Genome-wide association analysis was conducted on the salt-tolerance indices of phenotypic traits by employing the kinship matrix (K matrix) in a mixed linear model (MLM) to avoid the spurious marker trait associations (MTAs) caused by genetic background. In the present study, the *P* value indicated whether an SNP was associated with corresponding trait, and the *R*^2^ indicated the phenotypic variation explained by the markers. As Bonferroni–Holm correction (Holm, 1979) for multiple testing (α = 0.05) was too conserved and no significant MTA was detected with this criterion, markers with an adjusted -log_10_ (*P* value) ≥ 3.0 were selected as significantly associated markers (Gurung et al., 2014; Houston et al., 2014; Bellucci et al., 2015; Liu et al., 2017, 2019). Besides, Manhattan and Q–Q plot were drawn using CMplot package (Yin et al., 2020) implemented in R 3.5.3.

### Allelic Effects and Candidate Genes

Candidate genes were identified as all the genes located in LD block region around the significant SNP (±3 Mb based on LD decay analysis) of each important locus from the physical position of IWGSC^3^ (IWGSC v1.1). In the present study, alleles with positive effects on higher salt tolerance at seeding stage are referred to as “favorable alleles,” and those contributing to lower tolerance are “unfavorable alleles.” The peak SNPs for each locus were used to count the alleles frequencies and allelic effects. Regression analysis between favorable or unfavorable alleles and corresponding traits were conducted using Microsoft Excel 2019.

## Results

### Marker Coverage, Population Structure, and LD

**FIGURE 1:**
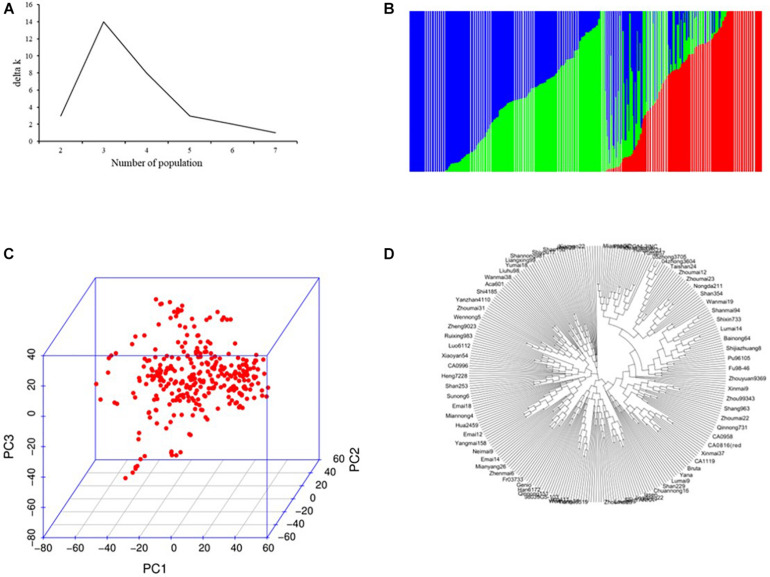
Population structure analysis of 317 wheat accessions. **(A)** Estimated ΔK over five repeats of structure analysis; **(B)** three subgroups inferred by structure analysis; **(C)** principal components analysis (PCA) plots; and **(D)** neighbor-joining (NJ) tree.

In total, 54,121 SNPs (14,063.9 Mb, 0.26 Mb per marker) was applied to GWAS for salt tolerance–related traits in 317 wheat cultivars (Supplementary Tables 1, 2). In total, 18,623 (34.41%), 21,091 (38.97%), and 14,407 (26.62%) markers were from the A, B, and D genomes, with 4,934.5, 5,179.0, and 3,950.4 Mb, respectively. The average genetic diversity for the whole genome was 0.356 (0.009–0.500; Supplementary Table 2). All the 317 accessions could be divided into three subgroups based on the population structure, PCA, and NJ tree analysis (Figure 1). Of these, the subgroup I carried 93 accessions and was dominated by Anhui, Henan, Shandong, and foreign cultivars; subgroup II included 89 accessions and mainly comprising varieties from Hebei, Shanxi, and Shandong provinces; most accessions (135) belonged to subgroup III and mainly from Henan, Shandong, and Sichuan province (Supplementary Table 1). The LD decay analysis indicated that the LD decay distance was about 3 Mb for the whole genome (Supplementary Figure 1).

### Phenotypic Variations for Salt Tolerance–Related Traits

The salt-tolerance indices for SDW, RDW, SFW, RFW, SH, RL, and K content (labeled as RSDW, RRDW, RSFW, RRFW, RSH, RRL, and RK, respectively) were estimated in the current study. The contents of Na and Na/K in shoot under salt stress were also assessed as salt-tolerance index. Continuous variation was observed across all traits with approximately normal distributions (Supplementary Figure 2 and Supplementary Tables 2–4). The result showed that RRL, RSH, RRFW, RSFW, RRDW, RSDW, and RK contents ranged from 0.31 to 0.9, 0.62 to 1.03, 0.18 to 1.05, 0.26 to 0.78, 0.17 to 0.94, 0.41 to 0.94, and 0.58 to 1.32, respectively (Table 1). Meanwhile, the contents of Na and Na/K under salt stress ranged from 24.67 to 88.10 mg/g DW and 0.33 to 1.58, respectively. It indicated a wide range of variation in each salt tolerance–related trait. ANOVA for salt tolerance–related traits revealed significant differences (*P* ≤ 0.01) among genotypes (G; Supplementary Table 4), suggesting that much of the phenotypic variation was derived from genetic factors. However, the broad sense heritability (*h*^2^) could not be calculated because of the phenotype evaluated only in laboratory.

**TABLE 1:** Phenotypic variation of salt-tolerance index traits in the 317 wheat accessions.

**Trait**	**Minimum**	**Maximum**	**Mean**	**SD**	**CV (%)**	**Skewness**	**Kurtosis**
RSDW	0.41	0.94	0.62	0.09	12.96	0.43	0.76
RRDW	0.17	0.94	0.52	0.11	19.65	0.31	0.97
RSFW	0.26	0.78	0.43	0.07	16.34	0.63	2.10
RRFW	0.18	1.05	0.47	0.12	24.55	0.66	1.71
RSH	0.62	1.03	0.79	0.06	7.25	–0.05	0.33
RRL	0.31	0.90	0.62	0.08	12.82	–0.13	0.80
RK	0.58	1.32	0.98	0.12	12.67	–0.24	0.26
Na (mg/g DW)	24.67	88.10	50.40	11.60	23.35	0.58	0.34
Na/K	0.33	1.58	0.74	0.23	30.93	0.84	0.61

*RSDW, ratio of shoot dry weight under salt stress and control; RRDW, ratio of root dry weight under salt stress and control; RSFW, ratio of shoot fresh weight under salt stress and control; RRFW, ratio of root fresh weigh under salt stress and control; RSH, ratio of shoot height under salt stress and control; RRL, ratio of root length under salt stress and control; RK, ratio of shoot K content under salt stress and control; and Na, shoot Na content under salt stress.*

*Values of minimum, maximum, and mean for each trait except Na represent ratio of each trait value between salt stress and control.*

*SD, standard deviation; CV (%), coefficient of variation.*

Pearson correlations among the salt tolerance–related traits (salt-tolerance indices) of seedlings under salt stress were calculated, and the correlation coefficients for nine traits are shown in Table 2. RSDW showed significantly negative correlation with Na and Na/K and positive correlation with the other traits. Pairwise positive correlation among six traits (RRDW, RSFW, RRFW, RSH, RRL, and RK) was observed. Notably, RSHs were uncorrelated with Na, Na/K, and RK in shoot, whereas Na and Na/K negatively correlated with all the other traits except RSH.

**TABLE 2:** Correlation coefficients among nine traits of seedlings under salt stress.

**Pearson correlation**	**RSDW**	**RRDW**	**RSFW**	**RRFW**	**RSH**	**RRL**	**RK**	**Na**	**Na/K**
RSDW	1.0	0.687**	0.777**	0.611**	0.393**	0.369**	0.163**	−0.269**	−0.272**
RRDW	0.687**	1.0	0.619**	0.832**	0.207**	0.469**	0.142*	−0.406**	−0.390**
RSFW	0.777**	0.619**	1.0	0.618**	0.395**	0.379**	0.211**	−0.485**	−0.467**
RRFW	0.611**	0.832**	0.618**	1.0	0.200**	0.490**	0.253**	−0.404**	−0.413**
RSH	0.393**	0.207**	0.395**	0.200**	1.0	0.304**	0.108	0.009	0.006
RRL	0.369**	0.469**	0.379**	0.490**	0.304**	1.0	0.107	−0.125*	−0.171**
RK	0.163**	0.142*	0.211**	0.253**	0.108	0.107	1.0	−0.408**	−0.606**
Na	−.269**	−0.406**	−0.485**	−0.404**	0.009	−0.125*	−0.408**	1.0	0.927**
Na/K	−0.272**	−0.390**	−0.467**	−0.413**	0.006	−0.171**	−0.606**	0.927**	1.0

RSDW, ratio of shoot dry weight under salt stress and control; RRDW, ratio of root dry weight under salt stress and control; RSFW, ratio of shoot fresh weight under salt stress and control; RRFW, ratio of root fresh weigh under salt stress and control; RSH, ratio of shoot height under salt stress and control; RRL, ratio of root length under salt stress and control; RK, ratio of shoot K content under salt stress and control; and Na, shoot Na content under salt stress.

**Correlation is significant at the 0.01 level. *Correlation is significant at the 0.05 level.

### MTA Analysis

**FIGURE 2:**
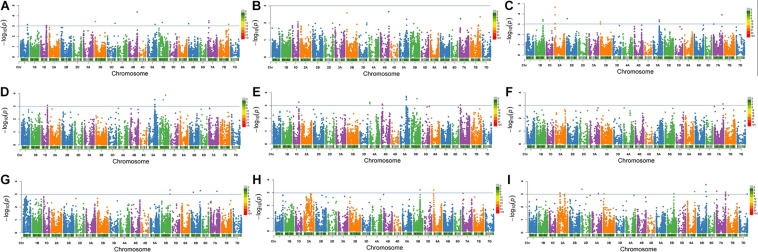
Manhattan plots for salt tolerance–related traits in 317 wheat accessions by the mixed linear model (MLM) in Tassel v5.0. **(A)** Relative shoot dry weight; **(B)** relative shoot fresh weight; **(C)** relative shoot height; **(D)** relative root dry weight; **(E)** relative root fresh weight; **(F)** relative root length; **(G)** relative shoot K content; **(H)** shoot Na content under salt stress; and **(I)** Na/K in shoot under salt stress. The −log_10_(*P*) values from a genome-wide scan are plotted against positions on each of the 21 chromosomes. Horizontal lines indicate genome-wide significance thresholds.

At *P* value of 0.001 (−log_10_ value of 3), a total of 37 significantly associated SNPs (MTAs) for eight salt tolerance–related traits including RSDW, RRDW, RRFW, RSH, RRL, RK, Na content, and Na/K were identified (Figure 2, Supplementary Figure 3, and Table 3). The 37 associated SNPs were distributed on 16 unique loci and located on chromosomes 1B, 1D, 2A (2), 4A, 5A, 5B (4), 6B, 7A (4), and 7B, respectively (Table 3), explaining phenotypic variation ranging from 6.3 to 18.6%. The number of loci found most in the A genome (8) and B genome (7), whereas only one locus was identified in the D genome (Table 3). Among these loci, four were detected for RSDW, four for RRDW, six for RRFW, five for RSH, one for RRL, two for RK, one for Na content, and five for Na/K (Table 3). Notably, nine loci (*IAAV8839*, *Kukri_rep_c68263_453*, *RAC875_c25567_1204*, *GENE-3440_199*, *Ku_c15213_388*, *Ku_c5191_340*, *BS00022442_51*, *BobWhite_c149_3064*, *and BS00071025_51*) were identified for two or more traits, proving the existence of pleiotropic regions. To add it, the quantile and quantile (Q-Q) plots of all the traits (Supplementary Figure 3) show that the expected −log10(*p*) value was close to the observed distribution (Supplementary Figure 2), indicating that the GWAS analysis through MLM (PC + K) was appropriate to locate MTAs related to salt tolerance in the germplasm under investigation.

**TABLE 3:** Loci for salt tolerance–related traits in 317 wheat accessions identified by Tassel v5.0.

**Trait^*a*^**	**Marker**	**Chr^*b*^**	**Pos^*c*^**	***P* value^*e*^**	**Marker *R*^2*f*^**	**QTL/gene^*g*^**	**Favorable allele^*d*^**
SH	*Excalibur_c112015_118*	1B	566.8	4.94E-04	8.5	Li L. et al., 2020	T
SH	*Ku_c956_1797*	1B	571.4	3.63E-04	8.4		C
RFW	*IAAV8839*	1D	430.4	5.51E-04	7.9	Li L. et al., 2020	T
RDW	*IAAV8839*	1D	430.4	8.46E-04	7.2		T
SDW	*IAAV8839*	1D	430.4	9.53E-04	7.1		T
SDW	*Ex_c18035_602*	1D	430.6	8.81E-04	7.1		G
SDW	*IACX1549*	1D	431.4	9.00E-04	7.3		A
SDW	*Kukri_c59519_352*	1D	431.7	9.61E-04	7.0		A
SH	*Tdurum_contig11803_306*	2A	36.0	1.22E-04	9.7		A
SH	*wsnp_Ex_c19556_28530243*	2A	36.1	2.19E-05	12.2		C
SH	*BobWhite_c26374_339*	2A	36.1	9.00E-04	7.6		T
Na/K	*Ku_c1217_312*	2A	182.3	8.09E-04	7.0		G
Na/K	*Ra_c22724_1137*	2A	182.3	9.14E-04	7.0		A
RFW	*JD_c21248_511*	4A	2.8	7.29E-04	7.5	Li L. et al., 2020	A
RFW	*BS00074614_51*	4A	3.0	5.61E-04	9.5		A
SH	*Kukri_rep_c68263_453*	5A	464.5	5.60E-04	7.9		T
SH	*Kukri_c17430_972*	5A	468.5	3.87E-04	8.6		C
RFW	*BS00027465_51*	5A	471.7	1.99E-04	9.0		G
RDW	*BS00027465_51*	5A	471.7	3.10E-04	8.4		T
RFW	*BS00027466_51*	5A	471.7	2.28E-04	8.9		C
RDW	*BS00027466_51*	5A	471.7	7.22E-04	7.5		C
RFW	*BS00022509_51*	5A	472.3	3.61E-04	8.5		A
RFW	*Kukri_c2326_1037*	5A	476.6	3.40E-04	8.4		T
RDW	*Kukri_c2326_1037*	5A	476.6	7.62E-04	7.3		G
RFW	*Kukri_c2326_995*	5A	476.6	2.39E-04	8.8		C
RDW	*RAC875_c25567_1204*	5B	273.1	3.13E-04	8.7		G
SDW	*RAC875_c25567_1204*	5B	273.1	4.46E-04	8.2		G
RDW	*GENE-3440_199*	5B	445.5	1.36E-04	9.5		A
RFW	*GENE-3440_199*	5B	445.5	2.99E-04	8.7		C
Na	*Ku_c15213_388*	5B	515.2	6.18E-04	18.6		T
Na/K	*Ku_c15213_388*	5B	515.2	9.25E-04	16.5		A
K	*Kukri_c16864_398*	5B	684.6	9.81E-04	7.3	Li L. et al., 2020	C
K	*Tdurum_contig65330_190*	5B	684.6	4.61E-04	8.4	Oyiga et al., 2018	G
Na/K	*Ku_c5191_340*	6B	668.8	1.84E-04	8.8		T
Na/K	*RAC875_rep_c69963_514*	6B	668.8	6.49E-04	7.4		C
K	*Kukri_rep_c101126_469*	6B	705.8	5.25E-04	8.4		A
Na/K	*BS00022442_51*	7A	30.1	5.93E-04	7.4		C
SH	*BS00062724_51*	7A	36.5	9.61E-04	7.2		A
SDW	*BS00040929_51*	7A	83.9	3.16E-04	8.6		G
SDW	*IAAV1971*	7A	84.7	4.65E-04	7.9		T
SH	*BobWhite_c149_3064*	7A	670.8	1.23E-04	11.4		C
RL	*wsnp_Ku_c19943_29512612*	7A	675.4	7.66E-04	7.2		C
Na/K	*IAAV6119*	7A	709.4	7.10E-04	7.2	Li L. et al., 2020	T
Na/K	*BobWhite_c46250_98*	7A	709.4	9.89E-04	6.7		C
SFW	*BS00071025_51*	7B	730.2	2.81E-04	6.3	Li L. et al., 2020	T
SDW	*BS00071025_51*	7B	730.2	6.81E-04	8.3		C

*^*a*^RSDW, ratio of shoot dry weight under salt stress and control; RRDW, ratio of root dry weight under salt stress and control; RSFW, ratio of shoot fresh weight under salt stress and control; RRFW, ratio of root fresh weigh under salt stress and control; RSH, ratio of shoot height under salt stress and control; RRL, ratio of root length under salt stress and control; RK, ratio of shoot K content under salt stress and control; and Na, shoot Na content under salt stress.*

*^*b*^Chr: chromosome.*

*^*c*^The physical positions of SNP markers based on wheat genome sequences from the International Wheat Genome Sequencing Consortium (IWGSC, http://www.wheatgenome.org/).*

*^*d*^Favorable allele (SNP) for traits is underlined.*

*^*e*^The *P* values were calculated by Tassel v5.0.*

*^*f*^Percentage of phenotypic variance explained by the MTA from the results of Tassel v5.0.*

*^*g*^The previously reported QTL or genes within the same chromosomal regions.*

### Relationship Between Salt Tolerance and the Number of Tolerance Alleles

**FIGURE 3:**
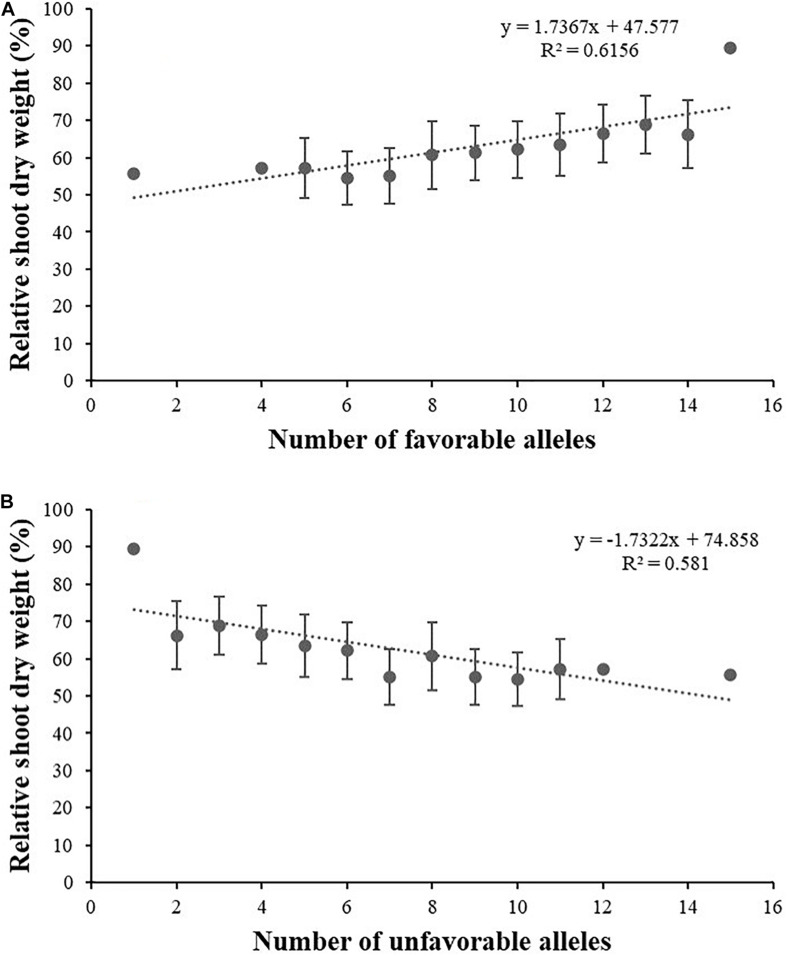
Linear regression between relative shoot dry weight and the number of favorable alleles **(A)** and unfavorable alleles **(B)** for salt tolerance.

Here, the RSDW of plants was used to stand for plant tolerance to salt stress (Szira et al., 2008). According to the relation between RSDW and other salt tolerance–related traits, favorable alleles for salt tolerance were counted as favorable alleles for RSH, RRDW, RRFW, RRL, RK, and unfavorable alleles for Na content and Na/K. To further understand the combined effects of alleles on salt tolerance, we examined the number of favorable alleles for salt tolerance in each accession (Supplementary Table 1). Interestingly, the number of favorable and unfavorable alleles in single accession both ranged from 1 to 15 (Supplementary Table 1). Linear regression analysis showed that RSDW displayed significantly positive correlation with total number of favorable alleles for salt tolerance (*R*^2^ = 0.616; Figure 3A) and notably negative correlation with number of unfavorable alleles (*R*^2^ = 0.581; Figure 3B). Thus, accessions with more favorable alleles and less unfavorable alleles were more tolerant to salt stress.

## Discussion

To date, many studies involved in physiological and molecular mechanisms on salt tolerance have been carried out in wheat, but the studies on identification of salt tolerance–related QTLs are limited (Ma et al., 2007; Genc et al., 2010; Xu et al., 2013; Asif et al., 2018; Oyiga et al., 2018). The information achieved is far from enough in salt tolerance improvement. Therefore, identification of much more QTLs/genes and further study were needed to improve salt tolerance in wheat. Association analysis is a highly efficient tool for the identification of QTLs for complicated quantitative traits. It makes possible to identify markers significantly associated with salt tolerance, thereby facilitating salt-tolerance breeding in wheat by MAS. The panel used in the present study showed a high genetic diversity of salt tolerance, consisting of landraces, advanced lines, and released cultivars from different ecological regions and countries (Table 1).

It has been reported that the biomass parameters of RSFW, RRFW, RSDW, and RRDW have been used together to determine salt tolerance under abiotic stress in previous studies (Zhao et al., 2010; Liu et al., 2018). RRL and RSH were also taken as important indicators to show plant salt tolerance in numerous studies (Hasan et al., 2017; Liu et al., 2018; Füzy et al., 2019). Moreover, the agronomic characters are always the primary targets in plant breeding (Zeng et al., 2003). In addition, shoot Na content and Na/K ratio were also considered as two of the key determinants for plant salt tolerance (Liang et al., 2018). Thus, these characters were used to identify QTLs for salt tolerance in the present study. Salt tolerance at seedling stage is important because the initial plant growth will affect the final production. Our study demonstrated large variation in various parameters among different wheat varieties at seedling stage under salinity stress (Table 1). The salt-tolerance index was employed for GWAS, as it is considered a reliable measure for assessing salt tolerance, which permits the control for background effects among different genotypes (Karan and Subudhi, 2012; Long et al., 2013; Kan et al., 2015). Significant genetic variations for the agronomic traits measured and shoot Na contents and Na/K ratio in this panel indicated the possibility of genetic improvement of salt tolerance (Yong et al., 2015; Oyiga et al., 2018).

The diverse panel consisted of 317 wheat accessions originating from 10 countries and could belong to three unique subgroups (Figure 1). The subgroups were generally consistent with geographic origins and pedigrees. For example, the CA1005, CA1062, and the CA1133 shared the common parent of Jing 411 in subgroup I; the released wheat cultivars Zhongmai 871, Zhongmai 875, and Zhongmai 895 were derived from Zhoumai 16, clustered with Zhoumai 16 in subgroup II; the Xiaoyan 54 and Xiaoyan 81 were clustered with Xiaoyan 6 in the subgroup III. To avoid spurious MTAs caused by population structure and kinship, MLM model (Q + K) based on Tassel v5.1 was adopted for association analysis (Zhao et al., 2007; Stich et al., 2008; Long et al., 2013). The LD decay is influenced by population structure, allele frequency, recombination rate, etc., and affects the precision of GWAS. Previous studies have been reported that LD decay in common wheat ranged between 1.5 and 15 cM using SSR, DArT, or SNP markers and various in landraces and cultivars (Liu et al., 2017), which showed a longer LD decay distance (Breseghello and Sorrells, 2006; Liu et al., 2017; Akram et al., 2020). Most of the accessions used in our study originated from the released cultivars and the improved cultivars. In this panel, the LD decay distance was approximately 3 Mb for the whole genome, in accordance with the previous studies (Breseghello and Sorrells, 2006; Liu et al., 2017; Akram et al., 2020). The marker densities for the whole genome are higher than LD decay distance and thus highly reliable for detecting MTAs in the diverse panel according to Breseghello and Sorrells (2006).

Among 16 QTLs discovered, nine co-associated QTLs (*IAAV8839*, *Kukri_rep_c68263_453*, *RAC875_c25567_1204*, *GENE-3440_199*, *Ku_c15213_388*, *Ku_c5191_340*, *BS00022442_51*, *BobWhite_c149_3064*, *and BS00071025_51*) were detected for two or more traits, suggesting that the genetic mechanisms of salt tolerance are complex. During the past two decades, more than 50 QTLs/genes for salt tolerance–related traits in wheat were identified using linkage or association mapping. Munns et al. (2003) have identified two major genes for Na^+^ exclusion, named *Nax1* and *Nax2*. Of these, *Nax1* was located on chromosome 2A and was identified by fine mapping as an Na^+^ transporter of the HKT gene family HKT7 (HKT1;4; Huang et al., 2006), whereas *Nax2* was located on chromosome 5A and identified as HKT8 (HKT1;5; Byrt et al., 2007). A locus for Na/K was identified in chromosome 2AS (182.3 Mb) in this study and is different with the *Nax1*. Besides, one locus for K content was identified in the 5B chromosome. The flanking sequence of the SNP *Kukri_c16864_398* and *Tdurum_contig65330_190* was compared with the homologous gene sequence in 5B, and the result demonstrated that the locus identified in this study is different with *Nax2*. Six common loci were identified in the present study in comparison with the QTLs detected previously based on the physical map of IWGSC V1.0 and integrated map of Maccaferri et al. (2015). Li L. et al. (2020) have found 269 associated loci on all 21 chromosomes in wheat for salt-responsive traits based on GWAS in a diverse panel of 323 wheat accessions and 150 doubled haploid lines; these overlapped with *Excalibur_c112015_118* (RSH, 1B), *Ku_c956_1797* (RSH, 1B), *IAAV8839* (RRFW, RRDW, and RSDW, 1D), *Kukri_c16864_398* (RK, 5B), *BobWhite_c46250_98* (Na/K, 7A), and *BS00071025_51* (RSDW and RSDW, 7B) in the present study. Oyiga et al. (2018) have identified a hub locus on chromosome 5B was simultaneously associated with germination vigor and index of dry root weight under different salt stress conditions by GWAS corresponding to *Kukri_c16864_398* for RK content in our study. Also, this locus was identified for germination rate under salt stress by Li L. et al. (2020). The results confirmed that GWAS is a powerful and reliable tool for identification of complex quantitative genes. The alignment of several loci for salt tolerance–related traits with previous studies also serves as a validation of the accuracy and powerfulness of the GWAS. The remaining 10 loci located on chromosomes 2A (*Tdurum_contig11803_306* and *Ku_c1217_312*), 5A (*BS00027465_51*), 5B (*RAC875_c25567_1204*, GENE-3440_199, and *Ku_c15213_388*), 6B (*Ku_c5191_340*), and 7A (*BS00022442_51*, *BS00040929_51*, and BobWhite_c149_3064) are likely novel, which may contribute to uncover the genetic mechanism of salt tolerance, and provide more opportunities for MAS breeding.

To identify candidate genes for the loci of salt tolerance, the sequences of SNPs associated with salt tolerance–related traits were used to BLAST against the National Center for Biotechnology Information. In total, five candidate genes were identified (Table 4). The SNP marker *Tdurum_contig11803_306* on chromosome 2AL corresponded to kinase family protein with 2.89 Mb, which may play crucial role in plant responses to salt stress by regulating the hypersensitivity to Na^+^ and superfluous accumulation of Na^+^ (Liang et al., 2018). Marker *GENE-3440_199* on chromosome 5B corresponded to E3 ubiquitin-protein ligase-like protein with 1.96 Mb, which plays an important role in plant growth and development (Cyr et al., 2002; Craig et al., 2009). It has been reported that the E3 ubiquitin protein ligase was involved in the regulation of the development of shoot and roots under abiotic stress (Guerra et al., 2012; Wang et al., 2020). Marker *Ku_c5191_340* on chromosome 6B and *BS00022442_51* on chromosome 7A corresponded to transmembrane 9 superfamily member with 2.31 Mb and transmembrane protein with 2.98 Mb, respectively. Various transmembrane transporters, such as H^+^/glycerol symporters, Na^+^/H^+^ antiporters, and the *P*-type ATPases HwENA1/2, either directly or through the electrochemical driving force of the proton gradient to respond to the salt stress in plants (Montpetit et al., 2012). In addition, many *Nax1* and *Nax2* (*HKT1;4* and *HKT1;5*) genes, well known as Na^+^ transporter genes, have been cloned and demonstrated to contribute to leaf Na^+^ exclusion and salt tolerance in wheat (Byrt et al., 2007, 2014; Tounsi et al., 2016). *Kukri_rep_c101126_469* on 6B encoded leucine-rich repeat receptor-like protein kinase family protein with 1.32 Mb, which may trigger multiple physiological pathways (Montenegro et al., 2017).

**TABLE 4:** Candidate gene for salt tolerance–related traits.

**Marker**	**Candidate gene**	**Gene position**	**mRNA position**	**Annotation**
*Tdurum_contig11803_306*	*TraesCS2A01G079300*	chr2A:36037402-36042137	chr2A:36037725-36041668	Kinase family protein
*GENE-3440_199*	*TraesCS5B01G261000*	chr5B:445453118-445459346	chr5B:445453337-445459168	E3 ubiquitin-protein ligase-like protein
*Ku_c5191_340*	*TraesCS6B01G393800*	chr6B:668776400-668778663	chr6B:668776660-668778663	Transmembrane 9 superfamily member
*Kukri_rep_c101126_469*	*TraesCS6B01G441700*	chr6B:705756764-705758664	chr6B:705756764-705758664	Leucine-rich repeat Receptor-like protein Kinase family protein
*BS00022442_51*	*TraesCS7A01G060900*	chr7A:30062542-30064125	chr7A:30062602-30063750	Transmembrane protein

For crops, we are concerned with yield much more than other characters. Biomass yield is often taken as a salt tolerance–related indicator because of its permission of the direct estimation of economic return under salt stress (Munns and James, 2003). Thus, in this study, biomass was considered the most important parameter related to growth under salt stress at seedling stage. The number of favorable alleles showed a significant positive effect on RSDW by the linear regression analysis, suggesting that pyramiding of favorable alleles may favor plant salt tolerance. Nine of the 16 loci were identified for two or more traits and should be applicable for MAS breeding. Some accessions with higher salt tolerance and relatively more favorable alleles, such as Xinong 291, Lumai 14, Wengnong 5, and Bima 4, should be excellent germplasms for breeding.

## Conclusion

In this study, a GWAS for salt tolerance in a diversity panel was conducted with the 90 K SNP chip. In total, 16 loci explained 6.3 to 18.6% of the phenotypic variations, demonstrating that GWAS can be used as a powerful and reliable tool for dissecting complex traits in wheat. The markers and accessions identified in this study can be used as valuable markers and excellent parent materials for salt tolerance breeding. This study improves our understanding of the genetic architecture of salt tolerance in common wheat.

## Data Availability Statement

The data presented in the study are deposited in the (Supplementary Material) repository, accession number (317).

## Author Contributions

XQ designed the research, analyzed the physiology data, and drafted the manuscript. XQ, NZ, CX, and HL performed the experiment. JL did the GWAS analysis. JL, YQ, and WH revised the manuscript. All authors have read, edited, and approved the current version of the manuscript.

## Conflict of Interest

The authors declare that the research was conducted in the absence of any commercial or financial relationships that could be construed as a potential conflict of interest.

## Footnotes

1 http://www.capitalbiotech.com/

2 http://software.genetics.ucla.edu/admixture/

3 http://www.wheatgenome.org/

## Supplementary Material

Supplementary Figure 1 The LD decay for the 317 wheat accessions.

Supplementary Figure 2 The frequency distribution of salt tolerance–related traits in 317 wheat accessions. **(a)** Relative shoot dry weight; **(b)** relative shoot fresh weight; **(c)** relative shoot height; **(d)** relative root dry weight; **(e)** relative root fresh weight; **(f)** relative root length; **(g)** relative shoot K content; **(h)** shoot Na content under salt stress; and **(i)** Na/K in shoot under salt stress.

Supplementary Figure 3 Quantile–quantile (Q-Q) plot for salt tolerance–related traits in 317 wheat accessions by the mixed linear model (MLM) in Tassel v5.1. **(a)** relative shoot dry weight; **(b)** relative shoot fresh weight; **(c)** relative shoot height; **(d)** relative root dry weight; **(e)** relative root fresh weight; **(f)** relative root length; **(g)** relative shoot K content; **(h)** shoot Na content under salt stress; and **(i)** Na/K content in shoot under salt stress.

Supplementary Table 1 The 317 wheat accessions used in the genome-wide association study (GWAS) for salt tolerance–related traits and their origins.

Supplementary Table 2 Basic statistical analysis of SNP markers used for genome-wide association study (GWAS) of 317 wheat accessions.

Supplementary Table 3 The salt tolerance–related traits in 317 wheat accessions.

Supplementary Table 4 The analysis of variance (ANOVA) for salt tolerance–related traits.
