# Supplementary figures and images for: Genome-Wide Association Study Uncover the Genetic Architecture of Salt Tolerance-Related Traits in Common Wheat (Triticum aestivum L.)
Source: Front Genet. 2021 May 20;12:663941. doi: 10.3389/fgene.2021.663941 (PMC8172982; doi:10.3389/fgene.2021.663941)

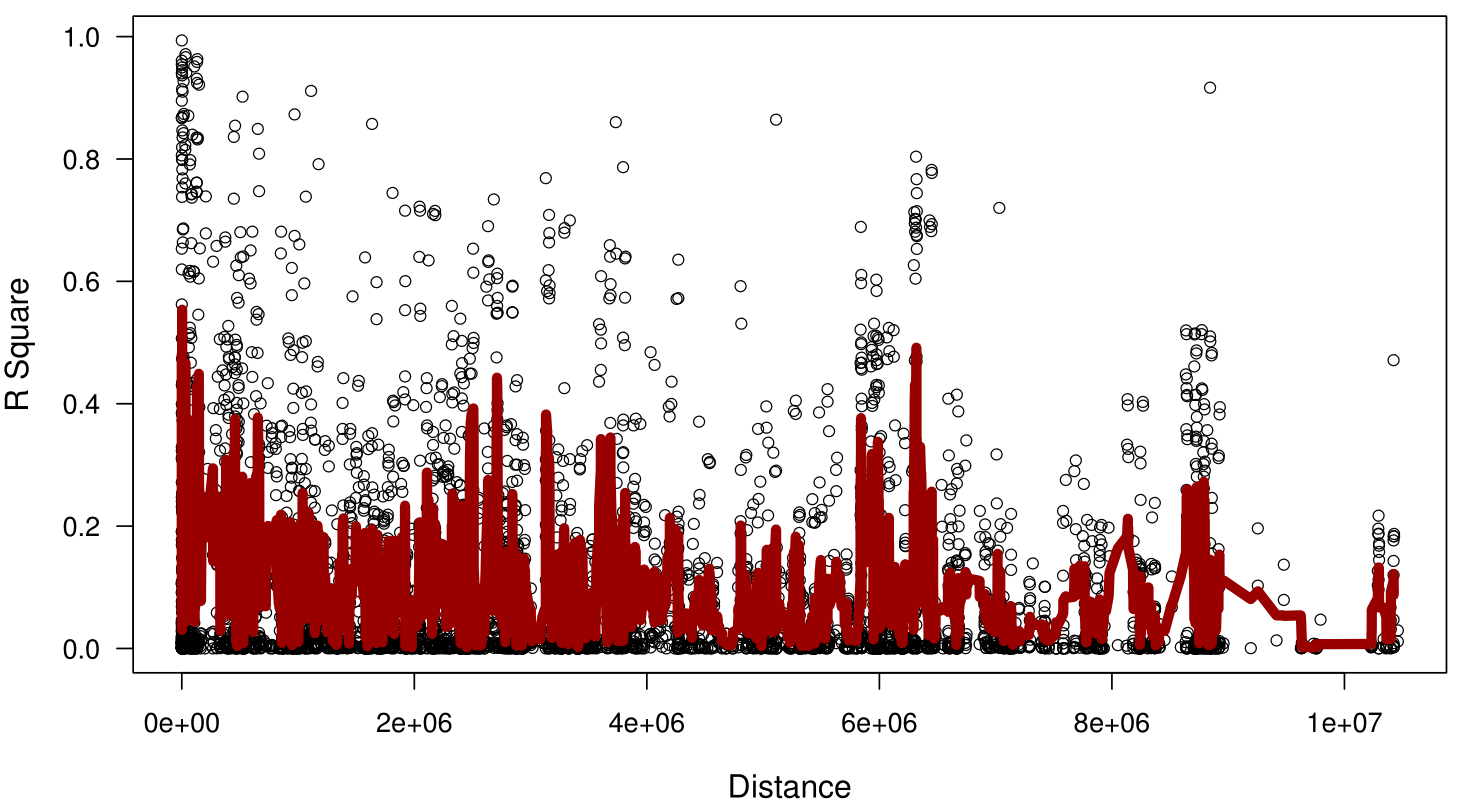

Supplement: Supplementary Figure 1 — The LD decay for the 317 wheat accessions. [file Image_1.PNG]

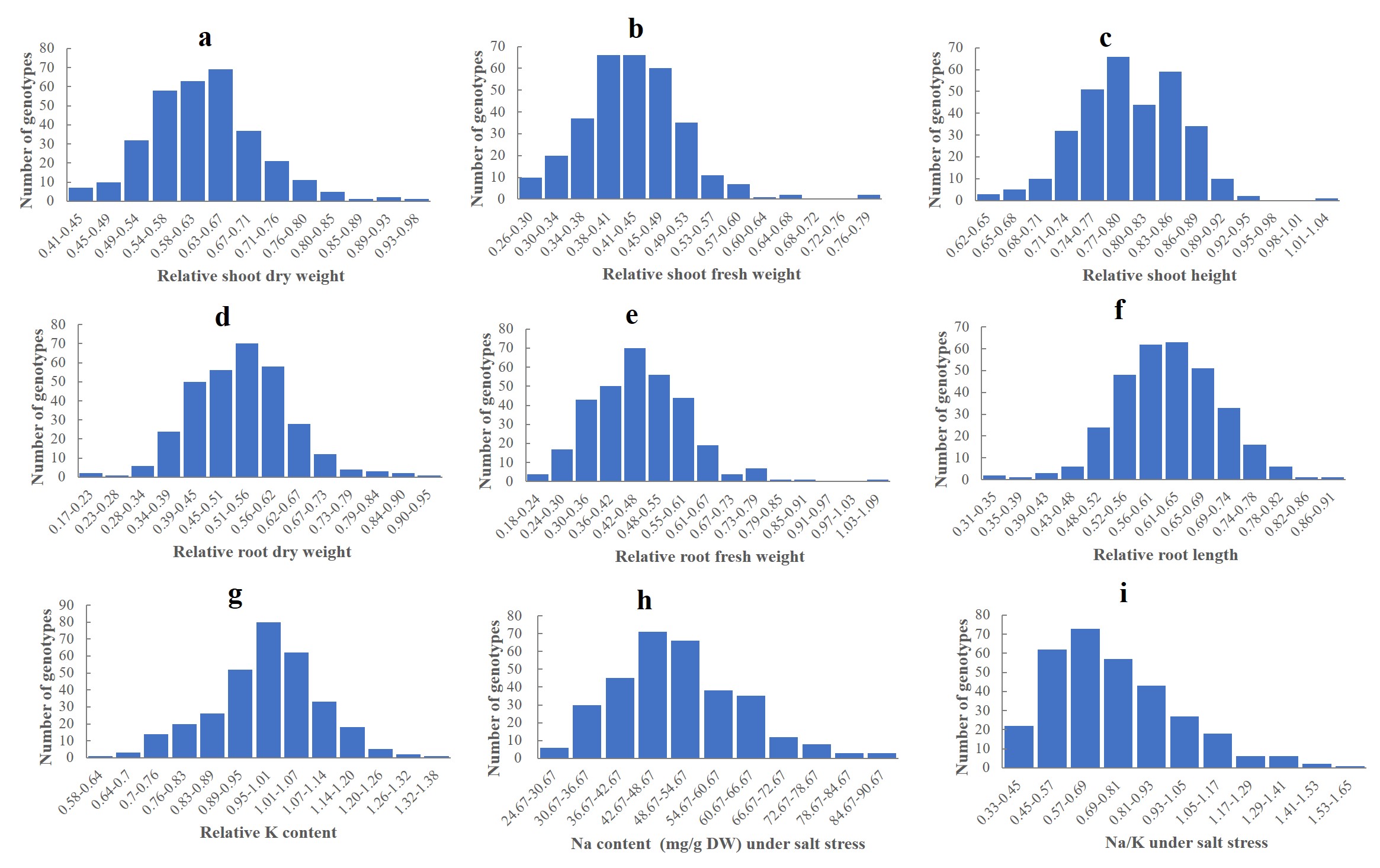

Supplement: Supplementary Figure 2 — The frequency distribution of salt tolerance–related traits in 317 wheat accessions. (a) Relative shoot dry weight; (b) relative shoot fresh weight; (c) relative shoot height; (d) relative root dry weight; (e) relative root fresh weight; (f) relative root length; (g) relative shoot K content; (h) shoot Na content under salt stress; and (i) Na/K in shoot under salt stress. [file Image_2.jpg]

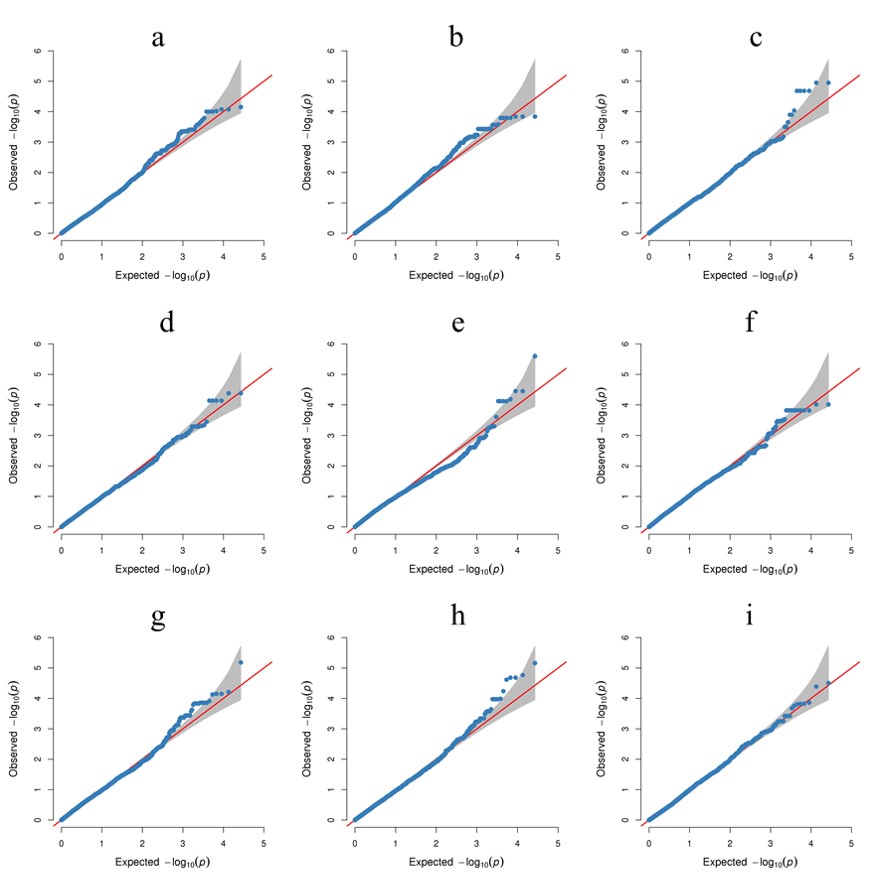

Supplement: Supplementary Figure 3 — Quantile–quantile (Q-Q) plot for salt tolerance–related traits in 317 wheat accessions by the mixed linear model (MLM) in Tassel v5.1. (a) relative shoot dry weight; (b) relative shoot fresh weight; (c) relative shoot height; (d) relative root dry weight; (e) relative root fresh weight; (f) relative root length; (g) relative shoot K content; (h) shoot Na content under salt stress; and (i) Na/K content in shoot under salt stress. [file Image_3.jpg]
