# Supplementary material for: Genome-Wide Association Study Uncover the Genetic Architecture of Salt Tolerance-Related Traits in Common Wheat (Triticum aestivum L.)
Source: Front Genet. 2021 May 20;12:663941. doi: 10.3389/fgene.2021.663941 (PMC8172982; doi:10.3389/fgene.2021.663941)
Supplement: Supplementary Table 4 — The analysis of variance (ANOVA) for salt tolerance–related traits. [file Table_4.docx]

**Table S4** The analysis of variance (ANOVA) for salt-tolerance related traits

| Source of  variation | Mean square | | | | | | | | |
| --- | --- | --- | --- | --- | --- | --- | --- | --- | --- |
|  | Na (mg/g DW) | RK | Na/K | RSDW | RRDW | RSFW | RRFW | RSH | RRL |
| Replicate | 545 | 312* | 128 | 107 | 169 | 188* | 231 | 105 | 120 |
| Genotype | 1269** | 546** | 319** | 306** | 238** | 576** | 303** | 129** | 121** |

**significant at *P* < 0.01

RSDW, ratio of shoot dry weight under salt stress and control; RRDW, ratio of root dry weight under salt stress and control; RSFW, ratio of shoot fresh weight under salt stress and control; RRFW, ratio of root fresh weigh under salt stress and control; RSH, ratio of shoot height under salt stress and control; RRL, ratio of root length under salt stress and control; RK, ratio of shoot K content under salt stress and control; Na, shoot Na content under salt stress
